# Editorial: Role of gut microbiota in diabetes mellitus and tumor immunity

**DOI:** 10.3389/fimmu.2023.1185080

**Published:** 2023-04-05

**Authors:** Jingxian Chen, Zhesheng Chen, Barkat Ali Khan, Kaijian Hou

**Affiliations:** ^1^ Department of Public Health and Preventive Medicine, Shantou University Medical College, Shantou, Guangdong, China; ^2^ Department of Pharmaceutical Sciences, College of Pharmacy and Health Sciences, St. John’s University, New York, NY, United States; ^3^ Drug Delivery and Cosmetic Lab (DDCL), Gomal Center of Pharmaceutical Sciences, Faculty of Pharmacy, Gomal University, Dera Ismail Khan, Pakistan; ^4^ Department of Endocrine and Metabolic Diseases, Longhu Hospital, The First Affiliated Hospital of Shantou University Medical College, Shantou, Guangdong, China; ^5^ School of Public Health, Shantou University, Shantou, Guangdong, China

**Keywords:** gut microbiota, diabetes mellitus, tumor, immunity, molecular mechanisms

Gut microecology is an important component of human health and environment. It’s composed of gut microbiota, gut epithelial cells, immune system, which forms gut mucosal barrier and act in energy metabolism. Gut microbiota plays an important role in the proper functioning of human organisms, it coevolves and symbiosis with humans by combating pathogenic bacteria, assisting in nutrient digestion, maintaining the integrity of the gut epithelia, and promoting immunological development. The steady state of the gut microbiota is closely related to human health and both external and genetic factors affect its composition and function. Thus, gut microbiota-mediated immunomodulatory effects play an important role in diabetes mellitus and tumor immunity. However, the causal relationship between an altered gut microbiota composition and diabetes mellitus or tumor immunity remains to be elucidated. Host immune responses are an extremely complex process and an in-depth exploration of the gut microbiota-host-disease relationship will be helpful to identify potential therapeutic targets for diabetes mellitus and cancer. We encourage the exploration of the etiology and factors influencing diabetes mellitus and tumor immunity from a gut microecological perspective with the aim of addressing these challenging questions. In this Research Topic, we aimed to explore gut microbiota profiles and the specific processes by which bacterial components, metabolites, and other mediators of gut microbiota interact with the host to affect the immunity of patients with diabetes mellitus or cancer.

This Research Topic included correlation analysis of gut microbiota composition with diabetes mellitus and tumor immunity. This review confirmed the differences in gut microbiota between healthy individuals and diabetic patients (Ye et al.). In addition, the differences in gut microbiota between type 1 diabetes mellitus and type 2 diabetes mellitus patients and the role of gut microbiota imbalance in the pathogenesis of diabetes patients, are discussed, especially with regard to the incidence of leaky gut syndrome, immune dysfunction, and metabolic disorders. They also summarized the progress made in developing microbial therapies to prevent and treat diabetes, especially the current status and application prospects of fecal microbiota transplantation in diabetes. Liu et al. expanded our understanding of the relationship between gut microbiota, an immune checkpoint inhibitor (ICI) response, and immune-related adverse events (irAE) occurrence. They studied the gut microbiota of patients who experienced a series of irAE in different cancers, particularly lung cancer; 1) with and without irAE; 2) with different severity of irAE; 3) with differences in microbiota composition between patients with and without irAE associated with colitis. Moreover, they explored the causal relationship between microbiota composition and immune-related colitis. Subsequently, their assessment of colitis and dynamic microbiota analysis led to more fundamental questions such as whether differences in microbiota cause immune-related colitis and whether immune-related colitis disrupts the gut microbiota. Huang et al. sequenced the ulcerated and intact skin of patients with diabetes and found that skin had significantly higher bacterial richness and diversity than diabetic foot wounds. The microecological balance of the skin was disrupted, and pathogenic bacteria replaced the original microbiota. It is suggested that commensal bacteria of intact skin may be important in maintaining microecological balance and preventing the development of diabetic skin ulcers.

This special issue also discussed gut microbiota-associated molecules that can affect host immune responses; Bao et al. reviewed such molecules from the perspective of traditional Chinese medicine (TCM), explaining why it can be a treasure trove of potential probiotics, and may shed light on the mechanisms of action underlying TCM successes in disease treatment. To further investigate the effect of TCM on different levels of gut microbial abundance. Lin et al. described gut microbiota characteristics of patients who were first diagnosed with diffuse large B-cell lymphoma (DLBCL), aiming to explore the correlation between immune indicators affecting patients and to clarify the role of the microecosystem-immune axis in the occurrence and development of DLBCL. In the future, it may be possible to improve the immune function of DLBCL patients by regulating the gut microbiota, increasing therapeutic efficacy and improving patient survival rates. Moreover, their study also revealed a correlation between changes in microbiota structure and host immunity, and this research suggests a better understanding of the specific mechanisms underlying the development of differential and dominant microbiota in DLBCL in future studies to identify new disease biomarkers and develop new therapeutic strategies.

This Research Topic also discussed the mechanisms whereby specific gut microbiota can affect the development of diabetes mellitus and tumor immunity; Yan et al. established and validated a diagnosis model based on microbial amplicon sequence variants markers for light chain(AL) amyloidosis in China, and initially explored the important role of gut microbiota in other types of amyloidosis. Their characterization of gut microbial communities in AL amyloidosis patients revealed that alterations in the abundance of certain gut microbial species may play a crucial role in regulating metabolic function and inflammatory responses. They further revealed the association between baseline gut microbiota and disease severity, response to treatment, and prognosis. Subsequently, they suggested that the effect of the gut microbiota on clonal plasma cell proliferation may be the focus of future studies. To further investigate this relationship between gut microbiome, immunity, and complications in diabetics, these authors established a diabetic cornea wound healing model in rodents (Bu et al.). They demonstrated that alterations in microvascular complications such as diabetic keratopathy and immune responses may potentially correlate with alterations in gut microbiota composition in diabetic patients. They were pleasantly surprised to find that these metabolic effects could be transferred to healthy lean individuals using a fecal transplant technique to produce a similar state of insulin resistance. Thus, the gut microbiota may be an important target in the treatment of metabolic syndrome and type 2 diabetes mellitus. The meta-analysis by Yan et al. showed differences in enterobacteriaceae in gestational diabetes mellitus (GDM) and non-GDM populations, and explored inflammation and possible immune mechanisms associated with the pathophysiology of this disease. The results showed specific changes in the composition of the gut microbiota in GDM patients. This suggests that we can provide bacterial targets for the prevention or treatment of GDM by rebuilding homeostasis of the gut microbiota.

The final contribution to this Research Topic centers on the molecular mechanisms whereby bacterial components, metabolites, and other mediators of gut microbiota, interact with the host immune system; Mao et al. used gut microbiota as a target to investigate differences in gut microbiota diversity between diabetic kidney disease (DKD) patients and non-DKD patients. Furthermore, the combined application of metagenomic and metabolomic approaches verified that a large number of metabolites produced by gut microbiota and circulating metabolites are key signaling molecules and substrates involved in metabolic reactions in the progression of DKD. In a surprising result, they confirmed the existence of an enteric-renal axis. The exploration of the molecular mechanism or pathway involved in DKD may be beneficial to the development of individualized prevention and treatment options. It is well known that short-chain fatty acids (SCFAs), a type of saturated fatty acid, are produced by the fermentation of the gut microbiome. Tao et al. discussed the role of gut microbiota imbalance in the pathogenesis of diabetic nephropathy under the influence of SCFAs. At the same time, it may also underlie immune response by gut microbiota imbalance in diabetic patients, which damages the structure and function of the kidney. They also discussed the role of SCFAs in regulating energy metabolism, the inhibition of inflammation, and oxidative stress, and the regulation immune response. Thus, they emphasize that SCFAs may be valuable in the diagnosis and treatment of DKD.

The importance of the gut microbiota is gradually being recognized by the field of microbiology due to the huge diversity seen in both its quantity and composition, and its ability to regulate metabolic organs. The reviews and original research in the present collection expand our understanding of the role of the gut microbiota and its metabolites as a critical endocrine organ, with a particular focus on the relationship between the gut microbiota and host diseases. We anticipate that future clinical validation of these findings and more detailed stratified analyses will identify novel biomarkers and disease-causing mechanisms that may permit prophylactic and/or therapeutic intervention.

## Author contributions

All authors listed have made a substantial, direct, and intellectual contribution to the work and approved it for publication.

